# Prevalence of apical periodontitis and quality of root canal fillings in population of Zagreb, Croatia: a cross-sectional study

**DOI:** 10.3325/cmj.2011.52.679

**Published:** 2011-12

**Authors:** Jurica Matijević, Tina Čižmeković Dadić, Goranka Prpić Mehičić, Ivica Anić, Mladen Šlaj, Silvana Jukić Krmek

**Affiliations:** 1 Department of Endodontics and Restorative dentistry, School of Dental Medicine, University of Zagreb, Zagreb, Croatia; 2 Private dentistry practice, Zagreb, Croatia; 3 Department of Orthodontics, School of Dental Medicine, University of Zagreb, Croatia

## Abstract

**Aim:**

To determine the prevalence of apical periodontitis and assess the quality of endodontic fillings in the population of the city of Zagreb, Croatia.

**Methods:**

A total of 1462 orthopantomograms from new patients at 6 different dental practices was analyzed during 2006 and 2007. The presence of periapical lesions was determined by using the periapical index score (PAI). The quality of endodontic fillings was assessed according to the filling length and homogeinicity. Data were analyzed using *t* test and ANOVA with Scheffe post-hoc test.

**Results:**

There were 75.9% of participants with endodontically treated teeth and 8.5% of all teeth were endodontically treated. Only 34.2% of endodontically treated roots had adequate root canal filling length, while 36.2% of root canal fillings had homogenous appearance. From the total number of teeth with intracanal post, 17.5% had no visible root canal filling. Using PAI 3 as a threshold value for apical periodontitis, periapical lesions were detected in 8.5% of teeth. Adequate quality of root canal fillings was associated with a lower prevalence of periapical lesions.

**Conclusion:**

We found a large proportion of endodontically treated teeth with apical periodontitis and a correlation between the quality of endodontic filling and the prevalence of periapical lesions. This all suggests that it is necessary to improve the quality of endodontic treatment in order to reduce the incidence and prevalence of apical periodontitis.

Apical periodontitis (AP) is a result of microbial contamination of periapical tissues that originates from a necrotic dental pulp or inadequately treated root canals (1-3). Diagnostic criteria for AP include the presence of symptoms and clinical signs during clinical examination and analysis of radiographs (periapical or panoramic). Radiographic analysis is important because AP in its chronic form is often overlooked and left untreated. Despite the relatively low risk of exacerbations (less than 5%), the influence of AP on remote organs and tissues persists even in its sub-acute chronic form (4). The outcome of endodontic therapy is assessed radiographically (5), but due to high availability and reliability, radiographs are also suitable for epidemiological studies. The prevalence of AP among adults in different populations is well documented in the literature, especially in the Scandinavian countries (6-11). The prevalence of AP positively correlated with age and this tendency will presumably increase because of an increase in dentate population (6). Multi-rooted teeth have a higher prevalence of AP than single-rooted teeth, especially molars (12). Factors that determine the outcome of endodontic treatment are length and quality of endodontic filling and post-endodontic restoration of the tooth (13-15). Nevertheless, epidemiological studies performed worldwide show that AP is present in 22-65% of root-filled teeth (14-17). The crucial factor for a successful healing of the periapical region is adequate root canal therapy with complete obturation of the root canal less than 2 mm from the external apical foramen (14-17). Another factor influencing the prevalence of AP is the quality of coronal restorations. Teeth with inadequate restorations were associated with greater occurrence of AP (8,18-20). So far, many studies on the prevalence of AP and its association with various factors have been done worldwide, but not one has been conducted in this part of Europe. Since epidemiologic data are essential for population health assessment, future health care planning, and funding, the aim of this study was to assess the prevalence of AP and the quality of root canal fillings in the population of the city of Zagreb, Croatia.

## Materials and methods

### Case selection

**Figure 1:**
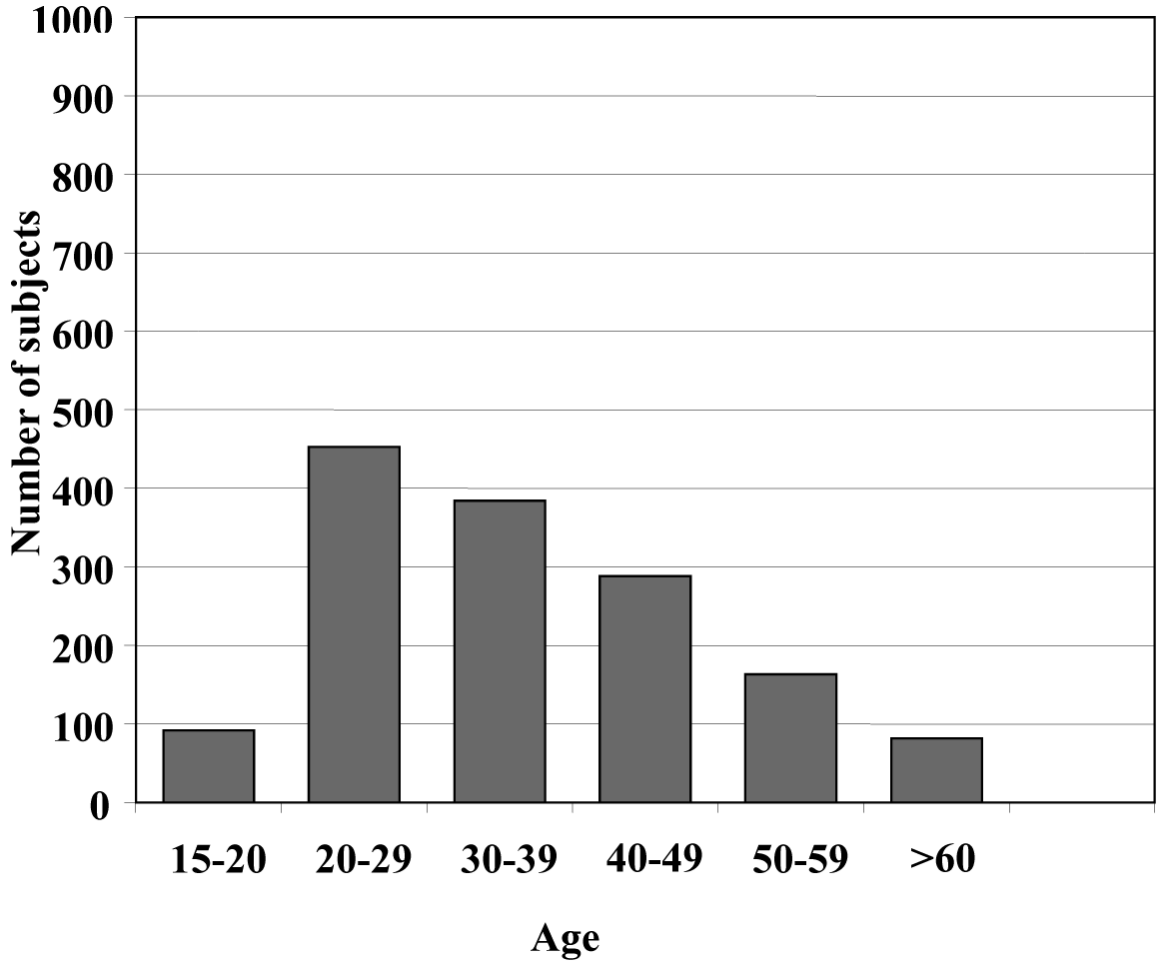
Age distribution of the sample.

The study was conducted during 2006 and 2007. A total of 2400 orthopantomograms (OPG) of patients attending source dental offices for the first time during a 6-month period were collected, shuffled, and every second was removed until 2000 remained. Out of 2000 OPGs, 1462 were included into the study, while 538 were discarded since they were multiple radiographs of the same patient (the chronologically first OPG was included in the study), had insufficient quality, or the patient was of inadequate age (age of 15 years was the minimum). The finally selected OPGs originated from five private dental offices in Zagreb with a contract with the Croatian Institute for Health Insurance (1320 OPGs) and the Department of Endodontics and Restorative Dentistry, School of Dentistry, University of Zagreb (142 OPGs). The study was approved by the Ethics Committee of School of Dentistry and every patient signed an informed consent for the usage of radiographs in the study. Participants were divided according to age and sex (Figure 1).

### Investigators

Investigators were one experienced clinician and one postgraduate student of endodontics and restorative dentistry, 2 persons in total. Calibration was performed prior to the study by determining the periapical index (PAI) according to Orstavik et al (21) and the quality of root canal filling on 100 periapical radiographs. Diagnostic reproducibility of investigators was established by a repeated analysis of 60 OPGs randomly selected from the sample (random number generator was used – 60 unique numbers per set, range 1-100) 6 months after the research had been completed. Cohen’s kappa values were 0.9 for AP and 0.81 for root filling quality.

### Radiographic analysis

OPGs were made with Orthopos model No. 5968573 D3 300 (Siemens, Munich, Germany) with constant current of 16 mA and 16-second exposure. Images were recorded on radiographic film (Kodak Lanex medium screens film; Eastman Kodak Company, Rochester, NY, USA) and processed in an automatic dark chamber processor (XR 24 Nova, Dürr Dental GmbH u. Co KG, Bietigheim-Bissingen, Germany) for 12 minutes.

OPGs were analyzed over a negatoscope (Dentsply R670425, Dentsply international, York, PA, USA) with 5 × magnification. Teeth were marked by dual digit system, following the recommendation of World Health Organization World Dental Federation (FDI) (22,23). Third molars were also included in the study. Absence of third molars was marked as a missing tooth.

### Endodontic status and quality of filling

The following categories were included into a status of the tooth:

• missing tooth – tooth lost, etiology unknown;

• endodontically treated tooth – tooth with root canal filling or material present only in the pulp chamber;

• post in the root canal with radiographically visible filling – non transparent shadow of metal intensity within the root canal with detectible root canal filling;

• post in the root canal without radiographically visible filling – non transparent shadow of metal intensity within the root canal without detectible root canal filling.

Endodontic status and technical quality of the root canal filling were determined according to Eckerbom and Magnusson (24).

Teeth with radiographically visible root canal filling and teeth with performed pulpotomy were included in the final calculation of endodontically treated teeth, as well as apicoectomied teeth with visible root canal filling and endodontically treated teeth with metal fragment in the root canal.

Length of the root canal filling of visible tooth roots was divided into five groups:

• group 1 – more than 5 mm from the radiographic apex;

• group 2 – between 3 and 5 mm from the radiographic apex;

• group 3 – less than 3 mm from the radiographic apex;

• group 4 – over the radiographic apex (overfilling);

• group 5 – radio-opaque material visible only in the pulp chamber (pulpotomy).

Homogeneity of the root canal fillings was divided into two groups:

• group 1 – complete obturation (homogenous appearance of the root canal filling);

• group 2 – incomplete obturation (voids and porous appearance of the root canal filling).

### Periapical status

Periapical lesions were classified using PAI, which was divided into five scores (21) as follows:

• score 1 – normal periapical appearance;

• score 2 – small changes in bone structures;

• score 3 – bone structure changes with small loss of minerals;

• score 4 – parodontitis with well visible radiolucent area;

• score 5 – advanced form of parodontitis with exacerbating appearance.

The threshold score for diagnosing AP was PAI score 3 and higher.

### Statistical methods

The data were entered into a prepared Microsoft Access database (Microsoft, Redmond, WA, USA). Before the analysis, data were exported to the Excel file format. Statistical analysis included descriptive statistics, *t* test, and ANOVA with Scheffe post-hoc test. We used SPSS, version 10 (SPSS Inc., Chicago, IL, USA) and *P* value was considered significant at 0.05. 

## Results

The total number of missing teeth was 8344. It represented 17.8% of 38 440, which was the maximum expected number of teeth (Table 1). In most cases, a participant had two missing teeth (165 participants – 11.28%) (Table 2). Upper right third molars were missing in 674 (45.5%) OPGs, upper left third molars in 643 (43.4%), and lower left first molars in 613 (41.4%). Lower left canines were missing in only 16 (1.1%) OPGs.

**Table 1:** Presence/absence of teeth and periapical status in patients from Zagreb

	No. (%) of teeth:							
	absent	present	root-filled	Periapical index score of all present teeth				
Teeth FDI index*				1	2	3	4	5
11-21	146 (4.99)	2778 (95.01)	313 (11.27)	2330 (83.87)	259 (9.32)	124 (4.46)	56 (2.02)	9 (0.32)
12-22	221 (7.56)	2703 (92.44)	311 (11.51)	2190 (81.02)	220 (8.14)	148 (5.48)	123 (4.55)	22 (0.81)
13-23	159 (5.44)	2765 (94.56)	239 (8.64)	2357 (85.24)	175 (6.33)	153 (5.53)	71 (2.57)	9 (0.33)
14-24	655 (22.4)	2269 (77.60)	295 (13.00)	1828 (80.56)	179 (7.89)	157 (6.92)	90 (3.97)	15 (0.66)
15-25	744 (25.44)	2180 (74.56)	381 (17.48)	1664 (76.33)	250 (11.47)	144 (6.61)	111 (5.09)	11 (0.50)
16-26	756 (25.85)	2168 (74.15)	344 (15.87)	1630 (75.18)	188 (8.67)	193 (8.90)	128 (5.90)	29 (1.34)
17-27	445 (15.12)	2479 (84.78)	174 (7.02)	2125 (85.72)	143 (5.77)	101 (4.07)	90 (3.63)	20 (0.81)
18-28	1300 (44.46)	1624 (55.54)	23 (1.42)	1526 (93.97)	38 (2.34)	30 (1.85)	20 (1.23)	10 (0.62)
31-41	75 (2.56)	2849 (97.44)	32 (1.12)	2743 (96.28)	51 (1.79)	16 (0.56)	33 (1.16)	6 (0.21)
32-32	46 (1.57)	2878 (98.43)	26 (0.90)	2791 (96.98)	43 (1.49)	12 (0.42)	26 (0.90)	6 (0.21)
33-43	40 (1.37)	2884 (98.63)	61 (2.12)	2771 (96.08)	54 (1.87)	26 (0.90)	27 (0.94)	6 (0.21)
34-44	231 (7.9)	2693 (92.10)	167 (6.20)	2453 (91.09)	112 (4.16)	60 (2.23)	61 (2.27)	7 (0.26)
35-45	512 (17.51)	2412 (82.49)	307 (12.73)	2001 (82.96)	152 (6.30)	131 (5.43)	112 (4.64)	16 (0.66)
36-46	1174 (40.15)	1750 (59.85)	338 (19.31)	1202 (68.69)	120 (6.86)	152 (8.69)	229 (13.09)	47 (2.69)
37-47	660 (22.57)	2264 (77.43)	218 (9.63)	1831 (80.87)	140 (6.18)	126 (5.57)	140 (6.18)	27 (1.19)
38-48	1180 (40.36)	1744 (59.64)	50 (2.87)	1531 (87.79)	92 (5.28)	49 (2.81)	67 (3.84)	5 (0.29)
Anterior	687 (3.92)	16 857 (96.08)	982 (5.83)	15182 (90.06)	802 (4.76)	479 (2.84)	336 (1.99)	58 (0.34)
Premolars	2142 (18.31)	9554 (81.69)	1150 (12.04)	7946 (83.17)	693 (7.25)	492 (5.15)	374 (3.91)	49 (0.51)
Molars 1 and 2	3035 (25.95)	8661 (74.05)	1074 (12.40)	6788 (78.37)	591 (6.82)	572 (6.60)	587 (6.78)	123 (1.42)
Molar 3	2480 (42.41)	3368 (57.59)	73 (2.17)	3057 (90.77)	130 (3.86)	79 (2.35)	87 (2.58)	15 (0.45)
Total	8344 (17.84)	38 440 (82.16)	3279 (8.53)	32973 (85.78)	2216 (5.76)	1622 (4.22)	1384 (3.6)	245 (0.64)

*Fédération dentaire international, World Dental Federation (23).

**Table 2:** Distribution of participants according to the number of missing teeth

Number of missing teeth	No. (%)
0	211 (14.4)
1	119 (8.1)
2	165 (11.3)
3	106 (7.3)
4	147 (10.1)
5	107 (7.3)
6	92 (6.3)
7	80 (5.5)
8	76 (5.2)
9	58 (4.0)
10	57 (3.9)
11	41 (2.8)
12	36 (2.5)
13	34 (2.3)
14	23 (1.6)
15	27 (1.8)
16	15 (1.0)
17	14 (1.0)
18	16 (1.1)
19	12 (0.8)
20	7 (0.5)
21	4 (0.3)
22	4 (0.3)
23	2 (0.1)
24	1 (0.1)
25	3 (0.2)
26	2 (0.1)
27	1 (0.1)
30	2 (0.1)
Total	1462 (100.0)

### Endodontic status

**Figure 2:**
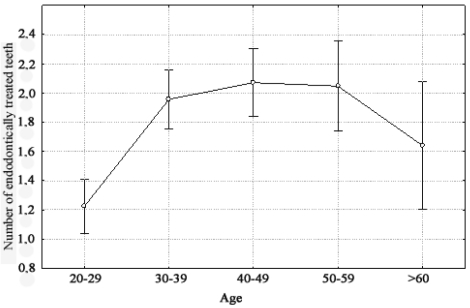
Association between age and the number of endodontically treated teeth.

From the total number of teeth, 8.53% were endodontically treated. Endodontically treated teeth were found in 1125 (75.9%) participants, while participants with only one endodontically treated tooth were most common (396 participants, 24.9%) (Table 1). There was a significant association between the age of patients and the number of endodontically treated teeth (ANOVA, F(4.1069) = 11.512, *P* = 0.001) (Figure 2). The most common endodontically treated teeth in the maxilla were second premolars and in the mandible first molars.

Out of 1100 teeth restored with an intracanal post, even 192 (17.5%) had no radiographically visible endodontic filling, but no significant difference in the prevalence and the severity of PAI scores of 3 and more between these two groups was found. Apicoectomied teeth without root canal filling and teeth with a fragment in the root canal but without root canal filling were seldom found (6 and 5 teeth, respectively in total).

Root canal filling was radiographically visible in 12.4% of roots. Radio-opaque material located only in the pulp chamber and/or at the root canal entrance was found in 10.7% of roots. Clinically acceptable length of the root canal filling of less than 3 mm from the radiographic apex was found in 1834 roots (34.2%). Length of the root canal filling of 3-5 mm from the radiographic apex was found in 1624 roots (30.3%) and length of the root canal filling of 5 mm or more from the radiographic apex in 1139 (21.2%) roots. Overfilling (root canal filling extending over the radiographic apex) was found in 196 (3.6%) roots. Homogenous appearance of endodontic filling was found in only 1942 (36.2%) endodontically treated roots.

### Periapical status

Periapical lesion determined with PAI 3 as a threshold value was present in 8.5% of teeth. Periapical index score 3 was found in 1622 (4.2%) teeth, while well marked and limited radiolucent periapical area (PAI 4) was found in 1384 (3.6%) teeth. Advanced form of periapical periodontitis with exacerbating appearance was found in 245 (0.6%) teeth (Table 1 and 3).

**Table 3:** Distribution of periapical index score >2 and ≤2 in all teeth and endodontically treated teeth

	No. (%) of teeth with periapical index score:			
	all		endodontically treated	
Teeth FDI index*	≤2	>2	≤2	>2
11-21	2589 (93.20)	189 (6.80)	197 (62.94)	116 (37.06)
12-22	2410 (89.16)	293 (10.84)	152 (48.87)	159 (51.13)
13-23	2532 (91.57)	233 (8.43)	111 (46.44)	128 (53.56)
14-24	2007 (88.45)	262 (11.55)	135 (45.76)	160 (54.24)
15-25	1914 (87.80)	266 (12.20)	210 (55.12)	171 (44.88)
16-26	1818 (83.86)	350 (16.14)	134 (38.95)	210 (61.05)
17-27	2268 (91.49)	211 (8.51)	77 (44.25)	97 (55.75)
18-28	1564 (96.31)	60 (3.69)	11 (47.83)	12 (52.17)
31-41	2794 (98.07)	55 (1.93)	18 (56.25)	14 (43.75)
32-32	2834 (98.47)	44 (1.53)	12 (46.15)	14 (53.85)
33-43	2825 (97.95)	59 (2.05)	26 (42.62)	35 (57.38)
34-44	2565 (95.25)	128 (4.75)	98 (58.68)	69 (41.32)
35-45	2153 (89.26)	259 (10.74)	151 (49.19)	156 (50.81)
36-46	1322 (75.54)	428 (24.46)	83 (24.56)	255 (75.44)
37-47	1971 (87.06)	293 (12.94)	74 (33.94)	144 (66.06)
38-48	1623 (93.06)	121 (6.94)	18 (36.00)	32 (64.00)
Anteriors	15984 (94.82)	873 (5.18)	516 (52.5)	466 (47.45)
Premolars	8639 (90.42)	915 (9.58)	594 (51.65)	556 (48.35)
Molars 1 and 2	7379 (85.20)	1282 (14.80)	368 (34.26)	706 (65.74)
Molar 3	3187 (94.63)	181 (5.37)	29 (39.73)	44 (60.27)
Total	35189 (91.54)	3251 (8.46)	1507 (45.96)	1772 (54.04)

*Fédération dentaire international, World Dental Federation (23).

**Figure 3:**
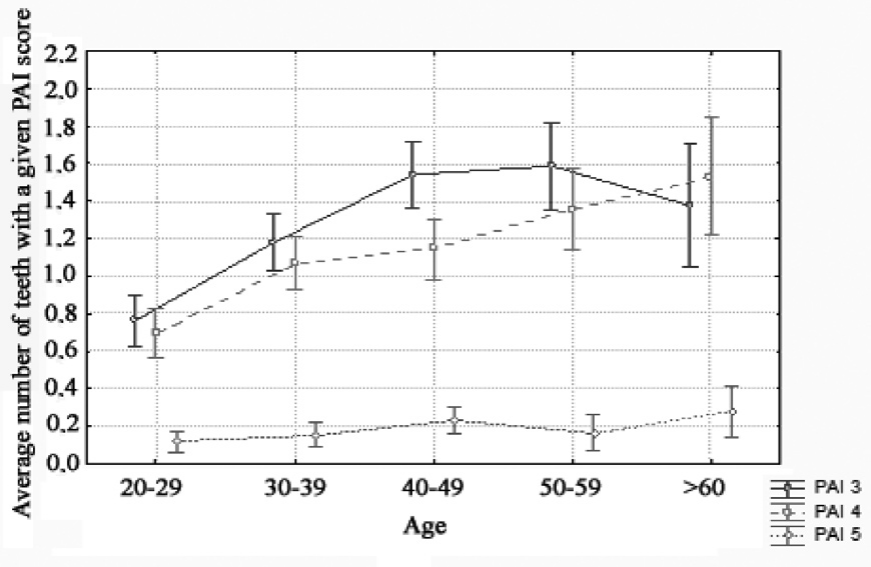
Association between age and periapical index score 3, 4, and 5.

ANOVA showed that the prevalence of PAI score 1 (healthy periapical structure) of endodontically healthy teeth and root-filled teeth that satisfy the length and obturation criteria decreased with age (F (8.2136) = 74.430, *P* < 0.05). *t* test for dependable variables showed a correlation between the quality of endodontic filling and the presence of periapical lesions (*P* < 0.05) in the same participant, ie, the PAI scores of teeth with root canal fillings that were well obturated and adequately long were significantly lower in the same participant. Association between age and PAI scores 3, 4, and 5 was also found (ANOVA, F(12, 2823.3) = 8.6188, *P* = 0.001) (Figure 3).

## Discussion

This study showed that 75.9% of participants in Zagreb, Croatia, had at least one endodontically treated tooth. Radiographically diagnosed AP was present in 8.5% of endodontically treated teeth.

The relationship between the quality of endodontic treatment and the prevalence of AP was shown in a large number of epidemiologic studies (14-20). Besides giving an insight into the population disease burden, epidemiological studies are a relevant source of data for health care policy and planning of funding. They may serve as a starting point in planning of health care measures, but also in assessing the efficacy of the performed measures (25). Endodontic epidemiology shows the prevalence of endodontic failures in general practice, with a high possibility of variations between the regions (26). Such studies have not been conducted in Croatia and the surrounding countries, which presents a problem not only in general health care planning, but also in the assessment of requirements for endodontic specialist care in the population.

The number of female participants in this study was 900 and the number of male participants was 562. Such ratio was not uncommon when compared with the results of previous studies (27,28). The reason for it might be women’s increased awareness of oral health importance and increased level of oral health care (27). However, similar studies showed that gender ratio had no effect on endodontic and periapical status (17,27-29).

According to a large epidemiological study in the USA (30), the majority of endodontically treated teeth was retained in the jaws for a period of 8 years and most of endodontic treatment complications happened in the first three years. Many studies have shown a strong correlation between the quality of root canal filling and the presence of AP (13-15,18,20,31,32). Another important factor that affects the periapical status is the quality and integrity of coronal restoration (8,14-16,18,20). In order to determine the quality of root canal fillings and the presence of AP, we used OPGs. OPGs were considered adequate because of the lower levels of radiation, simplicity of use, and satisfactory reliability (33,34). Also, no differences were found between PAI and densitometry in the efficacy of determining apical pathology (5).

In comparison with Eastern European countries, population in our study had a lower percentage of endodontically treated teeth (8.5%) than the Lithuanian population (15.0% including pulpotomy and root canal fillings) and Polish population from Łódź (9.8%) (35,36) and a higher percentage than Turkish populations (5.3% and 3.3%) (27,37). The prevalence was lower than in Western European countries – Swedish population had 13.0% of endodontically treated teeth (9) and a French study reported a high prevalence of endodontically treated roots (22.7% of roots) (31). A 20-year follow-up study in Sweden showed that the percentage of root canal-treated teeth increased from 13.8% to 17.7% within 20-year period (10). At the same time, an urban Danish population had lower prevalence of endodontically treated teeth – 4.8% (7). This might be an indicator of better general oral health care, but also a result of increased availability of endodontic therapy. Interestingly, the prevalence of AP was also greater. Besides differences in oral health care organization and availability of endodontic therapy, variability of study results may also be explained by differences in study designs and targeted populations.

The quality of the root canal filling is estimated by two criteria: the length of the root canal filling from the radiographic apex and the appearance of the root canal filling in terms of compactness or presence of gaps between the root canal filling and root canal walls (23). Our study confirmed the results of some previous studies that showed that a large percentage of root canal fillings were of poor quality (16,20,30). Adequate obturation was found in 36.2% of endodontically treated roots, while root canal filling length reaching apex was found in 34.2% of endodontically treated roots. For instance, a French study found that 20.8% of treated roots had sufficiently long root canal filling (31). Even less satisfactory situation was found in Lithuania, where only 13.0% of endodontically treated teeth had filling near apex (35). The main cause of unsuccessful endodontic treatment identified in this study is overfilling, with PAI score 3 found in 70.0% of cases (35). Most of the studies also showed that the quality of endodontic treatment was related to a successful outcome (20,38,39,40).

In our study, periapical lesions were found in 8.5% of teeth, higher than in the majority of other studies, which found periapical lesions in 0.6% to 13% of teeth (11,29). However, the comparison may not be reliable due to the different populations and study concepts. Our study confirmed that the quality of root canal filling played an important role in determining the presence of AP. Generally, in the same participant adequately endodontically treated teeth had lower PAI score than inadequately treated teeth. Besides the quality of root canal filling, another factor is also very important: the quality of the coronal restoration. A previous study demonstrated that teeth with inadequate endodontic treatment had periapical lesions in 75.7% of the cases, while the teeth with inadequate coronal restoration had periapical pathosis in 80% of the cases (41).

This study showed a high percentage of teeth supplied with posts, but without radiographically visible root canal filling (17.5%), which represents a potential difficulty if retreatment is required. Interestingly, in the teeth with posts the difference in the prevalence of PAI scores larger than 3 between teeth with and without root canal fillings was not significant, which is a finding that deserves further attention and research. Despite the limitations of panoramic radiography and population from a limited number of dental offices, the results of this study indicate the necessity of endodontic treatment improvement in Croatia. This could be achieved through better health care planning policy and education, increase in the number of endodontology specialists, and education of doctors of dental medicine in general practice. Also, in order to obtain relevant data for the whole country, it would also be advisable to conduct a larger-scale endodontic status survey with inclusion of offices from all Croatian regions with even better randomization.
